# Supplementary material for: InBRwSANet: Self-attention based parallel inverted residual bottleneck architecture for human action recognition in smart cities
Source: PLoS One. 2025 May 27;20(5):e0322555. doi: 10.1371/journal.pone.0322555 (PMC12111635; doi:10.1371/journal.pone.0322555)
Supplement: Attachment — Submitted filename: Response Sheet (1).docx [file pone.0322555.s002.docx]

Reviewer #1:

1- In the abstract, the authors state that they selected specific datasets for their model evaluation, but it remains unclear why these datasets were chosen exclusively. Please justify this selection more comprehensively to clarify the model’s applicability and limitations.

Response: Thank you for guidance, the relevant portion is added as guided.

The HMDB51 and UCF 101 are frequently used as action recognition standard datasets. These datasets are chosen to allow for meaningful comparison with earlier research. UCF101 dataset has wide range of activity classes and HMDB51 has varied real-world behaviors. These features test the generalizability and flexibility of presented model. Moreover, these datasets define the evaluation scope within particular domain and guarantee relevance to real-world circumstances.

2- The abstract should also include the achieved percentage accuracy for each dataset evaluated and report on the time complexity of the proposed model. This addition will enhance the reader’s understanding of the model’s performance and computational efficiency.

Response: Thank you for your suggestion. It is added as per your guidance.

The ablation study demonstrated that a margin of error value of 70.1338 ±3.053 (±4.35%) and 82.7813 ±2.852 (±3.45%) for the confidence level 95%,1.960σx̄ is obtained for HMDB51 and UCF datasets respectively. The training time for highest accuracy for HDMB51 and UCF101 is 134.09 and 252.10 seconds respectively.

3- In the introduction section, the advantages of using a vision-based approach over other modalities, such as sensor-based methods, need to be discussed in more detail. This would help establish the rationale for choosing vision-based methods. Additionally, please cite recent related works to provide a robust context for your study, such as: <https://doi.org/10.1109/CVPRW63382.2024.00344>; <https://doi.org/10.1109/TII.2024.3431070>; <https://doi.org/10.1016/j.aej.2023.11.017>

Response: The introduction section is updated as per your guidance with all citations at appropriate positions.

Vision-based recognition system poses a solution that is based on non-contact. The detection is considered as scalable and can be utilized for real-world applications. However, physical attachment is necessary in sensor-based approaches and these can be invasive. Spatial and temporal feature information can be attained using vision-based approach. It makes possible to classify human actions and related contextual information. DCNN based methods uses vision-based approach to classify actions and activities under a number of various circumstances. The potential of vision-based approaches is further highlighted by the current research. The vision-based approaches proved the effectiveness of neural network based dual-stream architectures in addressing complex spatiotemporal features and low lighting issues [22]. A number of novel frameworks like slow-fast tubelet (SFT) perform very well in lesser light and infrared conditions [23]. The challenges specific to domain in action recognition during sports activities is highlighted. The domain specific action recognition needs more specialized datasets [24]. These finding motivates on developing a customized DCNN for action recognition by stressing the flexibility and elasticity of vision-based methods.

4- The contribution section should be refined to clearly compare your work with these recent papers. Highlighting unique aspects of your approach will strengthen the section.

Response: The comparison is added before contribution paragraph in introduction section as guided.

Newly customized DCNN presents an efficient and effective way to recognize human actions. Its applicability is wide spread due to the usage of benchmark datasets (i.e. HMDB51 and UCF101). The proposed approach does not concentrate on domain specific datasets like [24] hence, it guarantees wider comparison and pertinence. The proposed architecture can handle RGB-specific recognition of actions without relying on specialized preprocessing as it is done in case of infrared-based preprocessing [23]. In proposed approach, single stream method is proposed and it perform better on HMDB51 whereas method by [22] is a dual-stream methods and it works better on UCF101 dataset. In a nutshell, single-stream proposed DCNN holds a compromise between efficiency and accuracy, which makes it suitable for a number of tasks without extra complexity.

5- An algorithmic representation of the proposed framework should be included. This will provide readers with a clearer understanding of the method’s step-by-step process.

Response: Algorithmic representation of the methodology is added as per guidance in methodology section.

The overall flow of the methodology is represented in given below algorithmic formulation.

Algorithm: Proposed Methodology

Step1: Selection of HMDB51 and UCF101 datasets

Step2: Customized DCNN with 6 number of inverted residual blocks

Step3: implementation of self-attention mechanism.

Step4: Model is trained on selected datasets

Step5: Features are extracted.

Step6: Multiple classifiers are used to classify features into relevant classes.

Step7: Performance measures are used to validate the proposed model.

6- Replace Figure 9 with a concise table that outlines the main module’s input and output.

Response: As per your guidance. The figure is now precise with relevant input and output.

7- Refine Figure 12 by representing the dataset class names with numbers, as demonstrated in the paper “<https://doi.org/10.1109/TBDATA.2024.3489414>” This will make the figure more straightforward and consistent.

Response: Thank you for guidance. Respective figure is improved.

8- In figure 13 the proposed model is not shown. Please adjust this figure.

Response: Figure is updated as guided

9- To further strengthen the discussion, compare your method with recent techniques, and delineate how your approach differs from existing methods. <https://doi.org/10.1109/TCE.2024.3373824>; <https://doi.org/10.1109/TBDATA.2024.3489414>

Response: Thank you for recommending. Both of the recommended papers are compared in introduction section

The transformer-based network in combination with MobileNetV3 and integration of self-attention is used to overcome limitations of computation in consumer electronics [25]. In proposed approach a general-purpose action recognition methodology is devised. The methodology takes care of extraction of important features and obtained competitive performance without using the complex transformers. Industrial HAR issues are resolved using squeeze bottleneck attention block (SBAB) and sequential temporal convolution network (STCN) [26]. Whereas, the proposed customized DCNN, provides a simplified and effective solution for RGB-based action recognition on HMDB51 and UCF101. The method maintains a balance between efficiency and computing efficacy without losing accuracy. So, the customized DCNN is a good choice for benchmark datasets and real-world applications.

Reviewer #2

This paper presents a deep learning method for detecting human action. The article is well organized and the proposed network structure is somewhat novel. Here are my comments:

1- The formatting of the formulas throughout the text is very confusing, e.g. some of the formulas are not centered and some are to the left.

Response: Thank you for your guidance. All equations are centrally aligned.

2- Figure 8 is very poorly viewed and could be drawn vertically or, instead of indicating the detailed block, replace it with an overall block box and then draw the detailed block separately.

Response: Thak you for your guidance. The figure is updated as per kind directions.

3- Figure 9 is not clearly visible, it should be a clear table, please redraw it.

Response: The figure is replaced with more concise explanation in tabular format.

4- The improvement over the best SOTA is very limited, and both datasets should be reproduced in all comparison methods.

Response: Thank you for your guidance, more recent work is added in comparison section of the manuscript.

In this section, a comparison of the proposed architecture with recent techniques using accuracy value is conducted. Table 3 compares SOTA techniques that employed HMDB51 and UCF101 datasets. In this table, authors in [31] used the HMDB51 dataset and obtained an accuracy of 65.90%. Authors in [28] obtained an accuracy of 70.23% using the HMDB51 dataset. Later on, Joudaki et al. [29] improved the accuracy of the HMDB51 dataset and reached 74.28%. The recent accuracy was further improved by Dastbaravardeh et al. [27], who obtained the highest value of 77.29%. In recent techniques proposed by Hussain et al.[25] and Altaf et al. [22] achieved 76.1428% and 78.6285% respectively on HMDB51. The proposed architecture obtained an accuracy of 78.80%, better than the current state-of-the-art techniques. For the UCF101 dataset, Ahmad et al. [34] recently obtained an improved accuracy of 91.79% which was previously 88.60% achieved by Wang et al. [35]. In a few recent works by **Hanzla et al.** [47] and Zhang et al. [49] on human action recognition using UCF 101 dataset, reported an accuracy of 86.90 and 88.10 respectively. The proposed architecture obtained improved accuracy on the UCF101 dataset of 91.80%, which shows this work's significance. Finally, some visual prediction results of the proposed architecture are shown in Figure 15. The original images are passed to the proposed architecture in this figure, returning a predicted output.

Table 3: Comparison with existing SOTA techniques

| Reference | Dataset | Year | Accuracy (%) |
| --- | --- | --- | --- |
| Dastbaravardeh et al. [27] | HMDB51 | 2024 | 77.29 |
| Amin et al. [28] | HMDB51 | 2019 | 70.30 |
| Joudaki et al. [29] | HMDB51 | 2024 | 74.28 |
| Abdorreza [30] | HMDB51 | 2023 | 71.13 |
| Yang et al. [31] | HMDB51 | 2022 | 65.90 |
| Hussain et al.[25] | HMDB51 | 2024 | 76.1428 |
| Altaf et al. [22] | HMDB51 | 2024 | 78.6285 |
| Proposed | **HMDB51** | **2024** | **78.80** |
| Dave et al. [32] | UCF101 | 2022 | 82.40 |
| Shen et al. [33] | UCF101 | 2021 | 79.60 |
| Ahmad et al. [34] | UCF101 | 2023 | 91.79 |
| Wang et al. [35] | UCF101 | 2024 | 88.60 |
| Korban et al. [48] | UCF101 | 2024 | 85.50 |
| Hanzla et al. [47] | UCF101 | 2024 | 86.90 |
| Zhang et al. [49] | UCF101 | 2024 | 88.10 |
| Proposed | **UCF101** | **2024** | **91.80** |
